# Estimation of Neuromuscular Primitives from EEG Slow Cortical Potentials in Incomplete Spinal Cord Injury Individuals for a New Class of Brain-Machine Interfaces

**DOI:** 10.3389/fncom.2018.00003

**Published:** 2018-01-25

**Authors:** Andrés Úbeda, José M. Azorín, Dario Farina, Massimo Sartori

**Affiliations:** ^1^AUROVA Group, Department of Physics, Systems Engineering and Signal Theory, University of Alicante, San Vicente del Raspeig, Spain; ^2^Brain-Machine Interface Systems Lab, Miguel Hernández University, Elche, Spain; ^3^Chair in Neurorehabilitation Engineering, Department of Bioengineering, Imperial College, London, United Kingdom; ^4^Department of Biomechanical Engineering, University of Twente, Enschede, Netherlands

**Keywords:** brain-machine interface, muscle primitives, corticospinal mapping, linear decoders, gait rehabilitation, lower-limb exoskeletons

## Abstract

One of the current challenges in human motor rehabilitation is the robust application of Brain-Machine Interfaces to assistive technologies such as powered lower limb exoskeletons. Reliable decoding of motor intentions and accurate timing of the robotic device actuation is fundamental to optimally enhance the patient's functional improvement. Several studies show that it may be possible to extract motor intentions from electroencephalographic (EEG) signals. These findings, although notable, suggests that current techniques are still far from being systematically applied to an accurate real-time control of rehabilitation or assistive devices. Here we propose the estimation of spinal primitives of multi-muscle control from EEG, using electromyography (EMG) dimensionality reduction as a solution to increase the robustness of the method. We successfully apply this methodology, both to healthy and incomplete spinal cord injury (SCI) patients, to identify muscle contraction during periodical knee extension from the EEG. We then introduce a novel performance metric, which accurately evaluates muscle primitive activations.

## 1. Introduction

A brain-machine interface (BMI) is a tool that can translate brain activity into device control commands, thus enabling an alternative pathway for the brain to physically act upon the environment (Wolpaw et al., 2002). In a rehabilitation context, BMIs are conveniently combined with wearable robots such as exoskeletons (Contreras-Vidal et al., 2016). One of the main challenges is the ability of restoring ambulatory functions in paraplegic patients with neurological conditions including incomplete spinal cord injury or stroke (del Ama et al., 2012). Within this scope, the combination of BMIs and lower limb exoskeletons can may exploit the concept of neuroplasticity, i.e. linking descending neural commands and peripheral somatosensory feedback to promote the reorganization of central nervous system damaged pathways in charge of motor control (López-Larraz et al., 2016).

BMI-extracted neural commands, which encode user's motor intentions, should be subsequently translated into control commands to the exoskeleton in real-time during the rehabilitation procedure. Reliable decoding of motor intentions and accurate timing of the robotic device actuation is fundamental to optimally enhance the patient's functional improvement (López-Larraz et al., 2016). It has been shown that motor intentions can be detected from electroencephalographic (EEG) signals and used to trigger an ankle exoskeleton so that the assisted movement was perceived as voluntary (Mrachacz-Kersting et al., 2012).

Current research has also focused on the relationship between low-frequency cortical modulations and motor tasks. Slow-cortical potentials (SCPs) reflect shifts in the cortical electrical activity lasting from several hundreds milliseconds to several seconds (Birbaumer et al., 1990; Shibasaki and Hallett, 2006). An example of this paradigm are movement-related cortical potentials (MRCPs) (Jiang et al., 2006; Shakeel et al., 2015). SCPs are triggered naturally as a person commences or imagines the onset of a movement. Moreover, there have been studies proposing the use of global cortical activity to extract kinematic information of upper and lower limb movements. In these studies, kinematic parameters were directly decoded from the activity of larger regions of the scalp by applying linear decoders to SCPs for decoding both upper and lower limb joint movements (Bradberry et al., 2010; Presacco et al., 2011), sitting and standing states (Bulea et al., 2014), finger movements (Paek et al., 2014), and types of grasping (Agashe et al., 2015). Other studies have dealt with the characteristics of the performed movement, showing that hand kinematics are better decoded when continuous and linear movements are performed (Úbeda et al., 2015) and exploring the possibility of using them to classify reaching directions (Úbeda et al., 2017).

In general, the use of linear decoders applied to SCPs are subject of controversy. Mechanical artifacts strongly affect the EEG low-frequency range during cyclic motion activities. This is suggested to directly influence the reliability of these decoders (Castermans et al., 2014; Costa et al., 2016). Moreover, other studies show that performance is not statistically different from chance levels due to the inherent properties of linear regression (Antelis et al., 2013). As a result, there is general consensus suggesting that current techniques are still far from being systematically applied to an accurate real-time control of rehabilitation or assistive devices (Úbeda et al., 2017). Indeed, only a few attempts reported to have obtained a reliable real-time decoder of movement kinematics (Bradberry et al., 2011). This study has been as well criticized for the way results are assessed which lead to performance similar to chance level (Poli and Salvaris, 2011).

The present study seeks to establish a reliable procedure to be applied in future real-time environments. Previous works are based on a single macroscopic regression function to directly map neural activity into the emerging/desired limb kinematics. In this, a single regression function may not be sufficient to capture all intermediate neuro-mechanical processes, thus only partly representing the mechanisms underlying movement. We suggest a possible solution to these major problems that is based on the combined use of linear decoders (for extracting high-level neural information) and multi-muscle electromyography dimensionality reduction (for capturing the basic spinal primitives of muscle control). Motor primitives encode information of the neural drive and have shorter pathways with respect to the cortical activity. As a consequence, our proposed method may be intrinsically robust (better signal to noise ratio) because it enables reconstructing a shorter neuro-mechanical gap (from brain activity to spinal cord activity) and applies the regression to a lower dimensional space (low-dimensional muscle primitives). Importantly, primitives have a lower dimensionality than lower limb kinematics, i.e., 12 degrees of freedom are needed to control 2 legs but only 4 primitives are needed to represent lower limb locomotion. To explore this methodology, we propose a novel approach that consists of detecting knee extensions from SCPs through the decoding of EMG primitives extracted from the recorded activity of the quadriceps femoris group.

## 2. Materials and methods

### 2.1. Experimental setup

Four patients (P01–P04) (2 males and 2 females, age: 43.5 ± 12.4 years old) were recruited from the patients services at the National Hospital for Spinal Cord Injury in Toledo. Only adults with incomplete spinal cord injury (iSCI) lesion above D7-D8, with ASIA C or D were selected. All patients were able to maintain standing position and ambulate for 30 m without external assistance and had enough functionality and strength in the upper limbs to use a walker or crutches. Additionally, four healthy subjects (H01–H04) (3 males and 1 female, age: 33.5 ± 7.9 year old) participated in the study. This study was carried out in accordance with the recommendations of the ethical committee of the National Hospital for Spinal Cord Injury and Miguel Hernández University of Elche, with written informed consent from all subjects. All subjects gave written informed consent in accordance with the Declaration of Helsinki. The protocol was approved by both committees.

**Figure 1:**
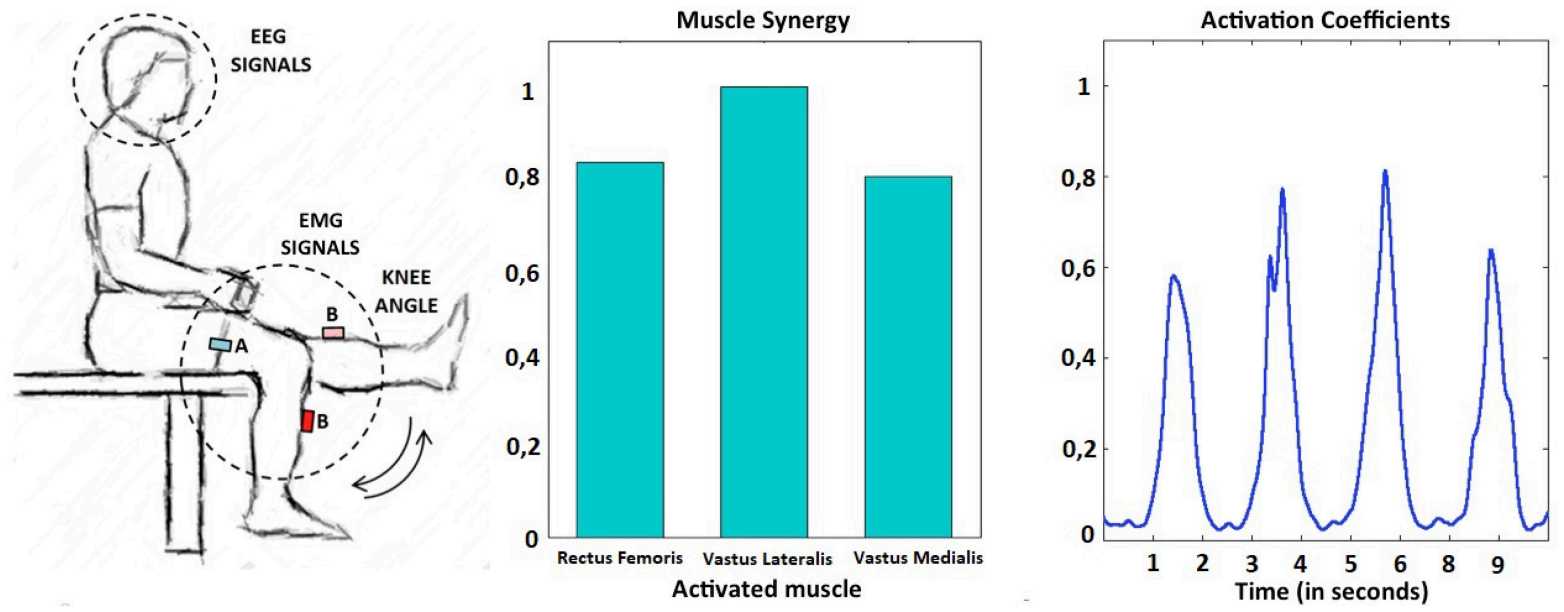
Experimental setup (left). Example of muscle primitive extracted during the performance of several self-paced extension movements: synergy weights (center) and non-negative activation coefficients (right). In this example, the subject performed four knee extensions in a period of 10 s.

Subjects seated comfortably on a chair and were asked to perform self-paced knee flexion-extension movements from full flexion (90°) to full extension (0°) (Figure 1, left). For each subject, data were recorded for 3 min, divided into 30-s runs with a 15-s rest period between them. Subject P04 only performed five runs due to fatigue. In the case of healthy subjects, the dominant leg was used to perform the movements. In the case of SCI patients, the movements were performed with the leg most affected by the lesion. All patients were capable of performing the knee flexion-extension movements, although more resting time between runs was given when necessary. During the performance of the knee flexion-extension movements, electroencephalographic (EEG) signals were recorded with two gUSBamp amplifiers (g.Tec, GmbH, Austria) at 1,200 Hz from 32 electrodes placed over the central and parietal cortex according to this distribution: FZ, FC5, FC1, FCZ, FC2, FC6, C3, CZ, C4, CP5, CP1, CP2, CP6, P3, PZ, P4, PO7, PO3, PO4, PO8, FC3, FC4, C5, C1, C2, C6, CP3, CPZ, CP4, P1, P2 and POZ. Subjects were asked to avoid blinks and head movements during each run. EMG signals were also recorded at 2,000 Hz from bipolar electrodes placed on 16 different muscles (Wave Wireless EMG, Cometa SRL, Italy). Additionally, knee angles were measured at 30 Hz using two inertial sensors (Technaid SL, Spain) placed on the thigh and on the leg.

### 2.2. Signal preprocessing

First, EMG signals were resampled to match EEG signal time stamps. Raw EEG signals were analyzed to reject blinks. To that end, sections of EEG data with abnormal amplitude were rejected. Afterwards, EEG signals were low-pass filtered with a zero-phase 2nd-order Butterworth filter (2 Hz). Finally, EEG data from each electrode were standardized by subtracting, for each time sample (*t*), the mean ($\bar{V}$) of the signal and dividing the result by the standard deviation (*SD*_*V*_) as shown in Equation (1). This standardization was computed for each individual run.

(1) $$EV[t]=\frac{V[t]-\bar{V}}{SD_{V}}$$

Raw EMG recordings were band-pass filtered (30–100 Hz), full-wave rectified, and low-pass filtered (6 Hz) using a zero-phase second-order Butterworth filter. For each subject and muscle group, the resulting linear envelopes were normalized with respect to the overall peak amplitude for that muscle. This was selected as the maximum value of a 50 ms moving-average window applied to the muscle linear envelopes across each recorded run (Gonzalez-Vargas et al., 2015).

### 2.3. Muscle excitation primitives

Non-negative matrix factorization (NNMF) (Lee and Seung, 2001) was performed for the set of consecutive extension cycles of each subject. Muscle activations are inherently non-negative. NNMF decomposes a data matrix (EMG activity) into a synergy matrix, W, and a command matrix, A, such that EMG = W^*^A, where the components of EMG, W, and A are all non-negative. During the knee flexion-extension exercise two primitives were identified, one active during the knee flexing phase and one during the knee extending phase. The knee extending phase was performed against gravity, resulting in pronounced extension primitives when compared to those extracted during the flexing phase. As a consequence, extension primitives (non-negative factors) were selected as major determinants of periodical multi-muscle contractions during the self-paced knee movements. An example of this behavior can be observed in Figure 1, right, where a representative subject performs four consecutive knee extensions. Each extension is commanded by an almost equal activation of all the muscles included in the quadriceps femoris group and can be explained by the extension primitive.

### 2.4. Linear decoder

To decode the muscle primitive in charge of knee extension, a multidimensional linear regression has been applied in a similar way to Úbeda et al. (2017) and according to the formula:

(2) $$x\left[t\right]=a+\sum_{n=1}^{N}\sum_{k=0}^{L}b_{nk}S_{n}\left[t-G*k\right]$$

where *x*[*t*] is the non-negative factor of the primitive at time *t* and *Sn* is the voltage measured at electrode *n*. *L* is the number of lags (past voltage samples), *G* is the gap between lags, *N* the number of electrodes and *a* and *b* are the weights of the linear regression. *N* corresponds to 16 (number of electrodes introduced in the decoder). *L* was fixed to 10, meaning that 10 time samples per electrode are selected to feed the decoder.

### 2.5. Automated EEG-based detection of periodical muscle contractions

#### 2.5.1. Electrode selection

**Figure 2:**
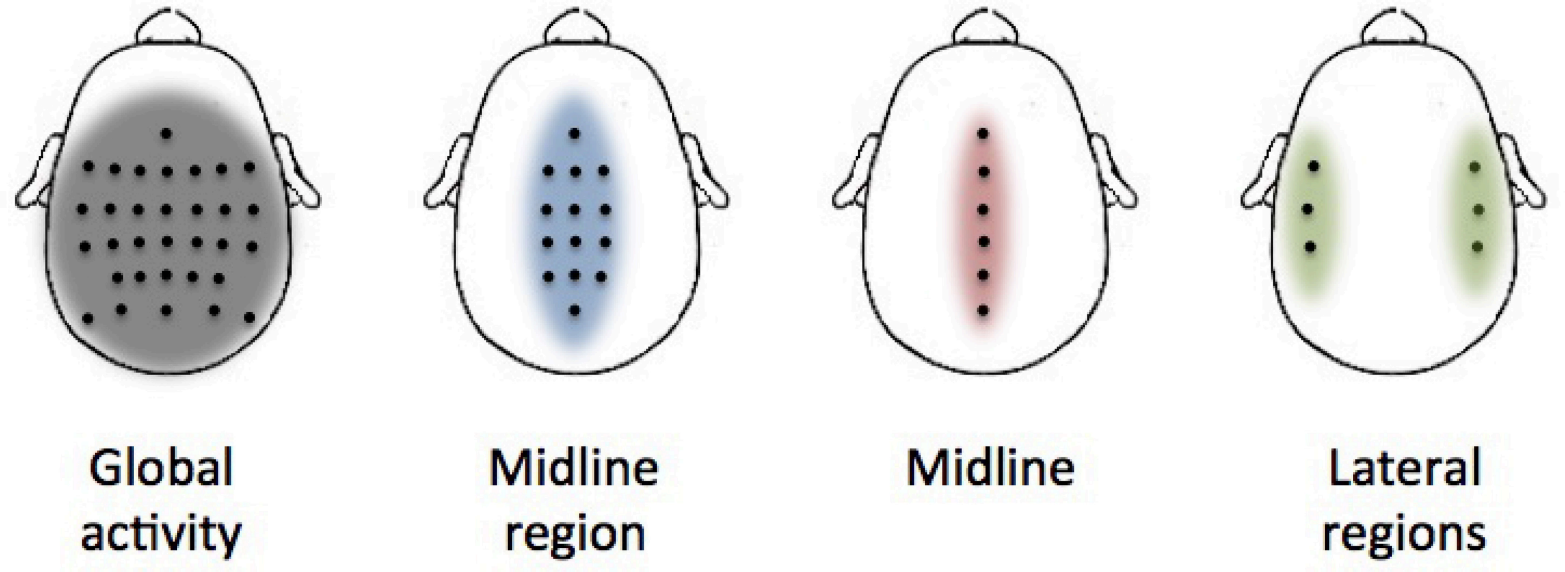
Electrode distributions used in the analysis. Each picture shows the location of each selected electrode (black dots) over the cortex. The colored area represents the approximate region of cortical activation.

Several distributions of electrodes have been evaluated to extract valuable information of the activation of different cortical regions during the performance of the movements (Figure 2). The first distribution covers all the recorded electrodes (global activity). This is in line with previous decoding studies where it is suggested that regions not located over the motor cortex have a significant contribution in decoding performance and, thus, they should not be discarded in the analysis (Agashe et al., 2015). However, under a classical electrophysiological basis, cortical modulations in charge of lower-limb motor control should be mainly located over the motor and premotor cortex midline (Brouwer and Ashby, 1990). Two distributions (midline region and midline) have been selected based on the assumption that motor cortex regions will provide a better performance than the activity of the whole cortex. The final distribution (lateral regions) has been selected to show if regions apparently not related to lower-limb motor activity have a significant contribution in the decoding performance.

#### 2.5.2. Decoding process

The proposed linear decoder has been applied for each electrode distribution. To improve decoding performance, the parameter G (gap) has been swept to evaluate a processing time interval from 100 ms to 2.5 s. Processing time interval has been limited to 2.5 s to minimize the effect of previous cycles in the decoding process. For each subject, a cross-fold validation (6-folds) has been applied (5-folds in the case of subject P04 who only performed 5 runs). For each fold, the training data was used to compute the weights of the linear regression that are then applied to the test data to obtain the decoded non-negative factors. We computed the Pearson correlation coefficient between the real and decoded primitives for each testing fold and reported the performance in terms of average correlation. All electrode distributions have been then compared to select the one with higher performance. From the selected distribution, the processing time interval with the higher correlation has been fixed for further analysis.

#### 2.5.3. Significance analysis

Shuffled data have been used as input to assess if the decoding accuracy was above chance levels. Shuffled data was obtained by randomly mixing trials of real cortical data and the associated non-negative factors to keep the temporal structure of the EEG signals in a way similar to Agashe et al. (2015). Shuffled data were filtered and standardized in the same way as the actual experimental data. Shuffled data decoding coefficients were computed for each subject with the previously selected best processing time interval for each electrode distribution. This means that, for each electrode distribution and subject, the cross-fold validation was applied to obtain a total of 96 correlation coefficients for healthy subjects and 92 for SCI subjects. This helps to avoid chance effects due to the stochastic nature of the process and also reduces the possible bias of a particular electrode distribution or subject.

#### 2.5.4. Identification of muscle contractions

The EEG-decoded muscle primitive was then compared to the one extracted from EMGs. To that end, peaks of maximum contraction were computed for both the original and decoded signal to obtain similarity metrics. Peaks were detected by looking for downward zero-crossings in the first derivative that exceeded a slope threshold and an amplitude threshold. The slope threshold was fixed to a very low value (10^−6^) while the amplitude threshold was fixed to 0.3 in the case of the original primitive and 0.05 in the case of reconstructed primitives, which were usually decoded with lower amplitudes.

True positive rate (TPR) was computed as the number of positive matches between the peaks extracted from both signals divided by the total number of extracted peaks. Only reconstructed peaks, which were closer than *M* times the average peak-to-peak distance in the original signal, were considered as positive. Detection rate (DR) was computed as the number positive matches divided by the number of peaks extracted from the original signal. Finally, time shift (TS) was computed as the average time shift between all the positive extracted peaks and their corresponding peak in the original signal. For comparison purposes, all the similarity metrics were computed for three different values of the parameter *M*: 0.1, 0.25, and 0.5. Additionally, the previously generated shuffled data was processed in the same way and compared to real data to evaluate the significance of the identification.

## 3. Results

**Figure 3:**
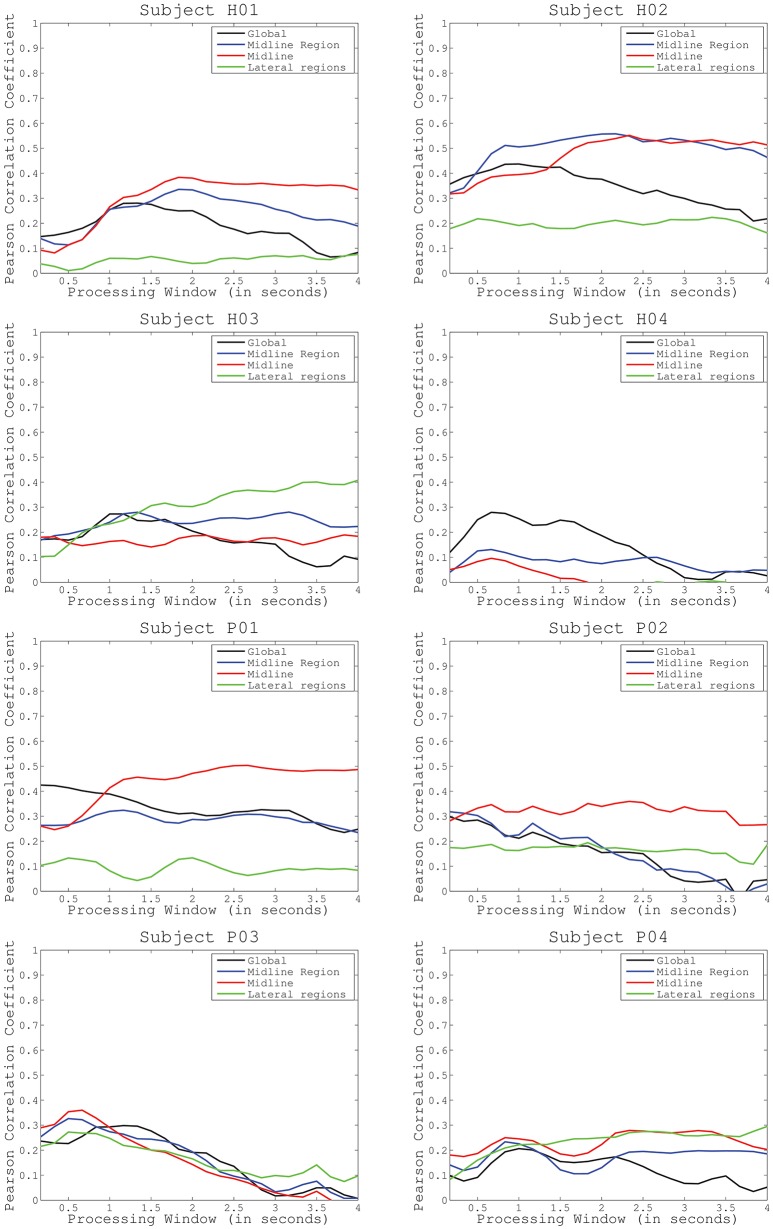
Average decoding performance for four different electrode configurations (global activity, midline region, midline, and lateral regions). Curves computed after sweeping the processing time interval (from 0.1 to 2.5 s) in the decoding protocol have been represented for healthy subjects (first and second row) and incomplete spinal cord injured patients (third and fourth row).

We performed three tests to evaluate the performance of the proposed methodology. The **first test** assessed decoding performance trends across subjects and conditions. Figure 3 shows average decoding performance across subjects and cortical regions. Each plot shows four curves that correspond to each preselected electrode distribution including: global activity, midline region, midline, and lateral regions. Each curve evaluates decoding performance for different processing time windows ranging from 100 ms to 2.5 s. Results show that decoding performance steadily increases from the minimum time window (i.e., 100 ms) and peaks at a subject-specific processing time interval (see Table 1). Then, decoding performance steadily decreases to levels similar to the starting point. This behavior is particularly evident for subjects H01, H02, H04, and for patient P03. Across all subjects and conditions decoding performance peaks approximately in the time frame 2–3 s (Figure 3). Subject H04 and patient P03 have however earlier peak decoding performance values, i.e., 0.66 s. Interestingly, lateral areas generally show worst decoding performance than global and midline areas. This is particularly visible in most of the subjects and patients, i.e., P01, P02, P04, H01, and H02. This is however not so clear in subjects H03 and H04 where lateral area decoding performance is most favorable.

**Table 1:** Selected electrode distribution and processing time interval (PTI) for each subject.

**Subject**	**Electrode distribution**	**PTI (s)**	**CC (mean ± STD)**
H01	Midline	1.83	0.38 ± 0.13
H02	Midline region	2.16	0.56 ± 0.12
H03	Lateral regions	2.50 (max)	0.36 ± 0.18
H04	Global activity	0.66	0.28 ± 0.10
P01	Midline	2.50 (max)	0.50 ± 0.17
P02	Midline	2.33	0.36 ± 0.19
P03	Midline	0.66	0.36 ± 0.18
P04	Midline	2.33	0.28 ± 0.11

*The corresponding decoding performance in terms of correlation coefficient (CC) is also shown*.

**Figure 4:**
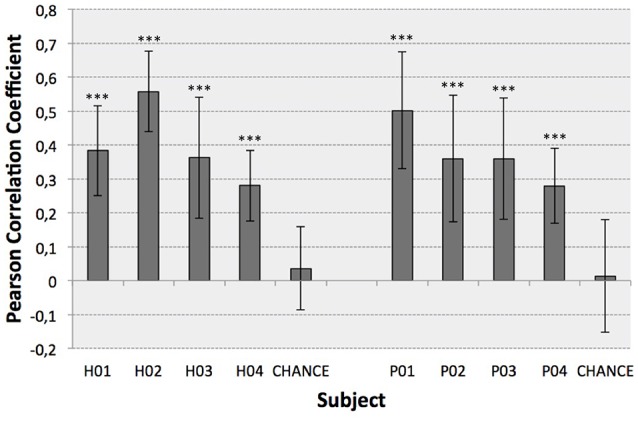
Optimal decoding performance for each subject (mean ± STD). Chance levels were also computed to infer the significance of the results (Wilcoxon Sum-Rank Test, ^***^p < 0.001).

**Figure 5:**
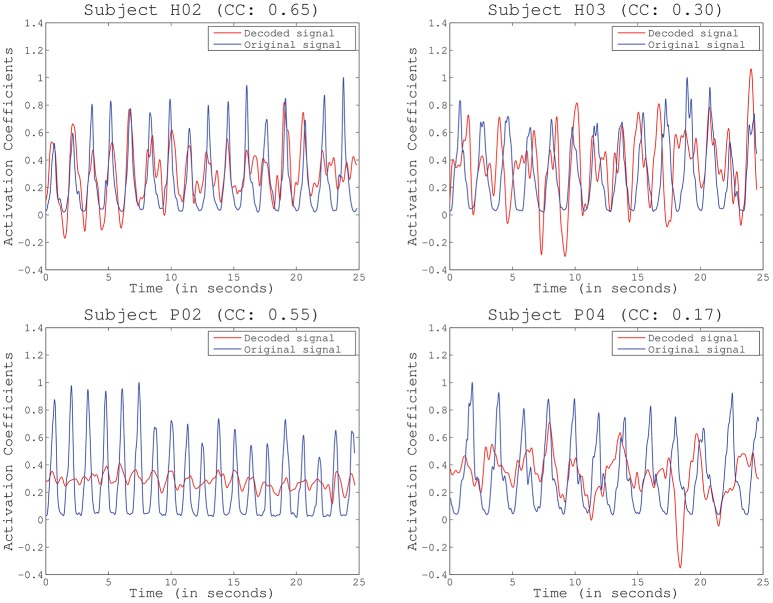
Example of reconstructed primitives for several representative subjects and folds, including the Pearson correlation coefficient (CC) obtained for the particular fold. Decoding performance does not depend on amplitude or baseline (subject P02) but decreases with increased phase delays (subjects H03 and P04).

The **second test** (Figure 4) identified the best decoding performance levels for each subject. These correspond to the processing time interval peaks in a particular electrode distribution (see Table 1). Additionally, chance levels (mean and STD) are represented for both healthy and SCI patients. The results obtained for all subjects are significantly different from chance levels (Wilcoxon Sum-Rank Test, *p* < 0.05). For most of the subjects and patients average decoding performance is >0.3 (subjects H04 and P04). Decoding performance for subjects H02 and P01 is >0.5. Interestingly, there is no significant difference between healthy and SCI subjects (Wilcoxon Sum-Rank Test, *p* > 0.05). Figure 5 shows a representative example of how decoding performance influences the behavior of the reconstructed signals. It presents the original muscle primitive (activation coefficients) and its reconstruction for 4 representative folds. Figure 5 (top-left and top-right) shows a similar behavior of the reconstructed signal despite the fact that decoding performance largely differs between these 2-folds. In the case of patient P02 (Figure 5, bottom-left), the decoding performance is high but the amplitude level mismatches between the original and the reconstructed signal. Finally, a poor reconstruction is shown in Figure 5 (bottom-right), where the reconstructed primitive does not correctly match the original signal.

**Figure 6:**
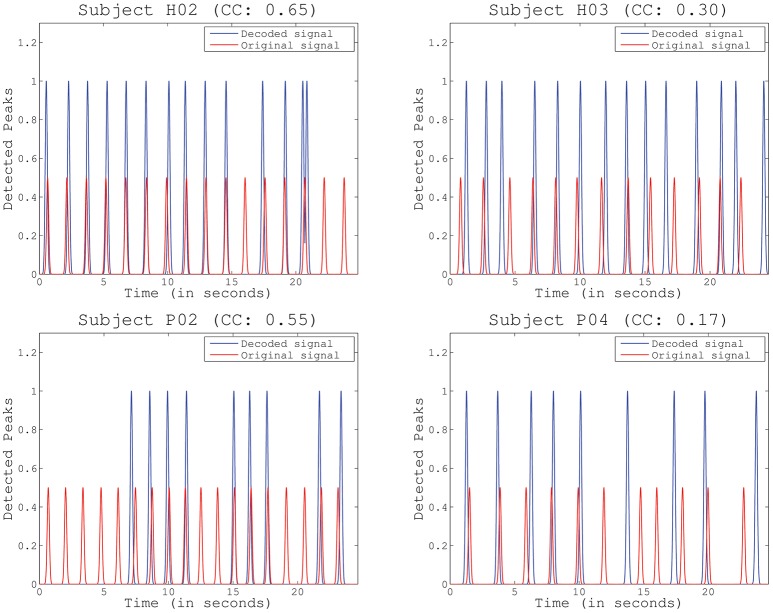
Example of identified peaks for several representative subjects and folds, including the Pearson correlation coefficient (CC) obtained for the particular fold. Scale differs between the decoded and the original peaks to improve visibility.

The **third test** assessed the ability of detecting periodical muscle contraction patterns based on reconstructed primitives and subsequently investigate how well they matched with the original primitives, i.e., those experimentally derived from EMG information. True positive rate (TPR), detection rate (DR), and time shift (TS) are presented (mean and STD) in Table 2 for each selected detection margin (M). From the table, we can see that TPR and DR increase with higher margins. Unsurprisingly, TS also increases with M. In this situation, the matching peaks rise in number, as there is a wider window of detection. TPR is generally consistent with previously obtained decoding performance. Subjects with higher decoding performance, such as H02 and P01, obtain the best results. Good TPR is also achieved for subjects H01 and P03 who, on the contrary, have lower correlation levels. Significant TPRs have been highlighted in the table after comparing them to chance levels (Wilcoxon Sum-Rank Test, *p* > 0.05). Interestingly, TPR computed for *M* = 0.5 is always above chance, while the remaining values are not always significantly different. To illustrate how the peaks are detected on both the original and the reconstructed signal, Figure 6 shows an example for the same representative subjects and folds shown in Figure 5. Peak detection performance on each of the graphs is clearly consistent with previous results on signal reconstruction. Figure 6 (top-left), with the best decoding performance of all four, presents a very good detection of original peaks (13 correct detections, 1 false detections, and 3 no detections). Figure 6 (top-right) also shows a high number of accurate detections (11 correct detections, 2 false detections and 3 no detection). The number of detected peaks is quite lower for Figure 6 (bottom-left), although peaks are identified with very good precision (9 correct detections, 0 false detections, 9 no detections). This is probably due to the bad scaling of the decoded primitive whose amplitude was comparatively lower than the rest. Finally, the fold with the worst decoding performance (Figure 6, bottom-right) shows, consequently, a quite low identification accuracy (6 correct detections, 3 false detections, 5 no detections).

**Table 2:** Similarity metrics (mean ± STD) for different detection margins (M).

**Subject**	***M***	**TPR (%)**	**DR (%)**	**TS (%)**
	0.1	42.07 ± 9.92	40.99 ± 12.72	0.08 ± 0.05
H01	0.25	74.19 ± 10.33	72.39 ± 18.28	0.19 ± 0.04
	0.5	87.48 ± 3.73	84.63 ± 14.32	0.26 ± 0.07
	0.1	60.51 ± 13.92	53.18 ± 15.22	0.06 ± 0.01
H02	0.25	88.08 ± 11.02	76.78 ± 13.31	0.11 ± 0.01
	0.5	98.81 ± 2.92	85.97 ± 7.97	0.15 ± 0.05
	0.1	25.09 ± 9.39	17.86 ± 11.68	0.09 ± 0.04
H03	0.25	76.25 ± 9.35	54.67 ± 28.88	0.24 ± 0.04
	0.5	100.00 ± 0.00	69.96 ± 31.47	0.32 ± 0.06
	0.1	33.07 ± 5.98	29.72 ± 7.18	0.10 ± 0.03
H04	0.25	70.99 ± 15.33	64.03 ± 17.89	0.20 ± 0.03
	0.5	94.86 ± 4.00	84.72 ± 10.56	0.31 ± 0.08
	0.1	40.99 ± 18.93	42.08 ± 22.10	0.10 ± 0.04
P01	0.25	81.19 ± 21.04	80.14 ± 22.80	0.27 ± 0.09
	0.5	90.95 ± 11.94	88.84 ± 12.20	0.34 ± 0.14
	0.1	31.08 ± 17.31	22.82 ± 9.98	0.09 ± 0.03
P02	0.25	65.48 ± 27.39	65.48 ± 27.39	0.16 ± 0.03
	0.5	95.57 ± 3.59	74.44 ± 14.46	0.25 ± 0.07
	0.1	35.30 ± 17.79	26.81 ± 19.33	0.08 ± 0.03
P03	0.25	80.14 ± 19.16	58.03 ± 30.94	0.18 ± 0.04
	0.5	93.10 ± 9.91	64.65 ± 27.05	0.22 ± 0.06
	0.1	39.72 ± 7.32	33.33 ± 7.42	0.11 ± 0.04
P04	0.25	67.61 ± 12.59	56.36 ± 9.07	0.19 ± 0.06
	0.5	94.11 ± 5.47	78.94 ± 9.59	0.37 ± 0.06

*True positive rates (TPR), detection rates (DR) and time shifts (TS) are presented for each subject and condition. In gray, conditions that are significantly different from chance levels (Wilcoxon Sum-Rank Test, p < 0.05)*.

## 4. Discussion

The main goal of this study was to obtain a robust way to translate brain signals into control commands provided to assistive devices such as robotic exoskeletons. For this purpose we proposed to identify motor primitives from SCPs. This enabled extracting high-level motor-related neural information and capturing the basic spinal primitives of multi-muscle control. As many motor processes are usually rhythmic, this methodology can provide a framework to identifiy periodical muscle activation from brain modulations that could be later applied to map full lower-limb mechanical information.

Current EEG-based continuous decoding techniques measure performance based on the cross-correlation between the original and the reconstructed signal. This correlation (Pearson correlation coefficient) is reported to be generally <0.4, which leads to difficulties in robustly translating the approach to a real-time assistive or rehabilitation scenario (Bradberry et al., 2010; Paek et al., 2014; Úbeda et al., 2017). One possible reason for low correlation metrics is the fact that current paradigms create direct mappings to body kinematics as a pure function of brain activity, thus bypassing all intermediate non-linear transformations, i.e., transmission pathways at the spinal and at the muscular level. As a consequence, important information may not be captured by a single macroscopic mapping. In our study we have decoded muscle primitives from brain activity instead of the direct kinematics of the lower limb. Results showed high decoding performance significantly above chance-level for all participants (Figure 4), with correlation coefficients being on average between 0.3 and 0.4, and reaching higher standard deviation values of up to 0.7. The obtained decoding performance was in line or higher than what was obtained in previous studies (Bradberry et al., 2010; Paek et al., 2014; Úbeda et al., 2017).

Moreover, our proposed approach offers the possibility to link decoded primitives to neuromusculoskeletal (NMS) models. In this context, it is not important to decode the exact shape and amplitude of activation primitives but just their timings. These would represent, in our formulation, the descending neural burst produced by the central nervous system (CNS) in the control of a group of muscles. In combination with modeling we propose in the near future to translate this burst into precisely timed mechanical function.

Our study resulted into three main findings: (1) decoding performance generally increases when only taking into account the information from motor cortex areas related to lower-limb movements and, thus, our approach is physiologically consistent with previous results of cortical motor control (Brouwer and Ashby, 1990), (2) we provide evidence that the processing time interval should be increased to achieve the optimal performance in the decoding process and that this time interval is generally in the proximity of 2 s which is consistent with the generation of anticipatory low-frequency potentials (Jahanshahi and Hallett, 2003); and (3) our proposed method to identify muscle contractions from the decoded primitives is less dependent on amplitude and phase variations compared to other correlation metrics such as the correlation coefficient.

This study was based on a small subject size so caution must be applied in the interpretation of results. However, it is worth stressing that our method proved to operate on individuals with spinal cord injury and with disrupted neuromusclar control. This itself is an important element providing initial evidence that our approach could be further extended and translated to larger clinical scenarios.

### 4.1. Evaluation of cortical involvement in the decoding

Cortical modulations in charge of lower-limb motor control are mainly located over the motor and premotor cortex midline (Brouwer and Ashby, 1990). For this reason, we have hypothesized that decoding performance of knee extension muscle primitives should increase if these particular areas are taken into consideration (test 1). We have compared decoding performance for different cortical regions to evaluate which distribution of electrodes increase the accuracy of the decoding (Figure 2). Closer inspection of the graphs in Figure 3 reveals that most of the subjects obtained a lower performance when taking into account lateral regions. This is consistent with our hypothesis, suggesting that regions not related to the lower-limb motor cortex have less influence in the decoding performance. For most of the subjects, the optimal electrode distribution is centered on the cortex midline (H01, P01–P04) or on the midline area (H02), which again suggests that these areas are more relevant when decoding lower-limb activity.

In contrast, in the specific case of subject H03, when the processing time interval increases, performance levels for lateral areas increased compared to the other distributions. Subject H04 also obtained better results from the global activity of the whole cortex. A possible explanation of this behavior may be found in the variability of the cortex modulations across subjects. Indeed, EEG analysis is highly subject-specific and, for singular individuals, certain regions, different from the motor cortex, could contribute to motor control as previously suggested in Agashe et al. (2015).

Another possible reason could be related to the presence of motion artifacts affecting the global activity of the whole cortex. Motion artifacts are a key limitation in the application of BMIs under ambulatory conditions, particularly during gait rehabilitation procedures (Costa et al., 2016). This fact could also explain why all the configurations showed a similar behavior in subjects P03 and P04. Even so, our results suggest that artifact influence, if any, is limited due to the experimental conditions: the experimenter permanently monitored head movement and the proposed task did not involve important movement transmission through the body affecting the head of the participants (subjects sitting during the performance of knee extensions). Also, the lower decoding performance that many subjects obtained for lateral areas in comparison to other configurations indicates that artifact activity is not dominating the decoded output, being more dependent on actual cortical modulations. However, a future application of the proposed methodology in more complex conditions, such as the decoding of locomotion, should consider this element, as it may hinder the translation of the BMI system to the technology level in a realistic environment.

### 4.2. Analysis of the processing time interval

Previous works used short processing time windows (i.e., 100 ms) in the application of linear decoders to SCPs, (Bradberry et al., 2010; Presacco et al., 2011; Úbeda et al., 2015). By using this approach, it is possible to obtain significant performance (in terms of signal-to-signal correlation) in the decoding of upper and lower limb kinematics. However, from the point of view of signal analysis, there is little variation of the signal amplitude in such a short time window, as the signals of interest are previously filtered below 2 Hz. This is even more critical in the case of EEG modulations, where the signal to noise ratio is particularly low. Our proposed approach provides robustness in this aspect. As a result, we could enlarge the processing time interval (i.e., increasing it up to 2.5 s), thus enabling information extraction from larger low-frequency EEG modulation windows.

The analysis of the processing time window revealed that decoding performance peaks were associated to larger processing time intervals, i.e., generally between 2 and 3 s (Table 1). Our results showed that longer processing time intervals not only carried more information of low-frequency modulations, but also have electrophysiological consistency, e.g., previous studies reported anticipatory SCPs initiating around 2 s prior to movement onset (Jahanshahi and Hallett, 2003). These findings are limited to the small size of the population (8 subjects) so the assessment of larger populations is necessary to validate this conclusion.

One of the limitations of our experimental setup is the requirement of periodicity of the knee extensions as the synergistic analysis generates primitives for cyclic movements. However, important functional tasks in daily life are cyclic, e.g., locomotion, stairs climbing, ramp ascending, etc. Therefore, our approach is expected to have important implications despite the cyclic constraint imposed by muscle primitives analysis. Indeed, this fact can explain why, for particular subjects and channel distributions, decoding performance curves do not peak (for instance, subject H3 for lateral regions or P1 for midline area) (Figure 3). If the processing time interval is longer than one cycle, cortical modulations responsible for previous cycles can sum their influence into the decoding performance. To minimize this effect, we limited the selected processing time interval to 2.5 s. We also believe that a longer resting period between extensions (instead of continuous movements) will provide a better analysis of the proper processing time interval. In this sense, further evaluation should analyze how well this method adapts to pauses or absence of the periodical activity. This is a critical aspect to be considered in future experiments that assess similar single-joint lower limb movements as well as those related to human locomotion.

### 4.3. Identification of muscle contractions

It is worth stressing that current correlation metrics may be limited in determining the true performance of our proposed system. Correlation is invariant to scale and location (baseline) but very dependent on phase. This can be clearly seen in our results. High correlation coefficients could be obtained from a very good reconstruction (Figure 5, top-left) or with important differences in amplitude (Figure 5, bottom-left). On the contrary, a low correlation did not always translate into a poor reconstruction, as it happened in Figure 5, top-right, where the reconstructed signal was only slightly shifted but accurate. Future work will determine the proper performance metric to be applied, which will eventually depend on the final goal of the study. In this sense, recent works have already discussed about the effects of applying different performance metrics (Spuler et al., 2015), from the more typical Correlation Coefficient (CC) applied in our study, to other methods such as Normalized Root Mean Squared Error (NRMSE) or Signal to Noise Ratio (SNR), among others.

As a result, our study employed additional metrics for evaluating decoding performance by extracting peaks in the reconstructed signals that match the original muscle contractions. This is more suitable for detecting neural bursts to feed NMS models, as this method solves some of the limitations of the previous performance metric, e.g., the high dependence on phase. As an example, the reconstructed primitive for Subject H03 in Figure 6, top-right, which had a quite low correlation, achieved a very high identification accuracy, while folds with high correlation kept a very low detection error (Figure 6, top-left and bottom-left). In the case of Subject P02, the bad scaling in the reconstruction increased no detections, but did not affect the number of false detections.

Our proposed identification method is still dependent on the tuning of internal parameters including the margin of detection (M) or the amplitude threshold (Section 2.5.4). When parameter L was increased, the true positive rate importantly increased (Table 2). This is somehow misleading and does not represent a proper identification of decoded peaks because of the already mentioned continuous periodicity of the knee extensions. In fact, a very wide margin can lead to the misidentification of many detected peaks that are not really close to one of the peaks in the original signal. On the other hand, short margins failed to detect most of the peaks. To evaluate this issue we have a applied a paired test between our identification results and chance levels showing that both low (*M* = 0.1) and high (*M* = 1) margins reduce the significance of the identification accuracy (true positive rate) and, that an average length of this margin (*M* = 0.5), which corresponds to half of the peak-to-peak distance in the original signal, is a more suitable tuning for parameter *M*. This tuning is a critical aspect in the timing of actuated gait-assisting devices in realistic scenarios.

### 4.4. Further application of corticospinal mapping

Our proposed procedure accurately extracts the activation onsets of muscle primitives and, thus, reduces the dimensionality of the decoding by directly mapping corticospinal transmission. Extracting muscle primitives from EEG signals may be more physiologically plausible than directly decoding joint kinematics as EMG extracted motor primitives encode alpha motor neuron discharges and have shorter pathways with respect to the cortical output.

Another important advantage of the proposed method is the reduction of dimensionality in the decoding procedure. In cyclic movements, such as locomotion, up to 12 different variables are needed to define movement, e.g., during gait, while with this procedure it is possible to reduce this output to just 4 primitives.

In addition, we evaluated new metrics that may be more suitable to trigger, for instance, an exoskeleton during gait assistance, as they are more sensitive to cyclic muscular activations. In this regard, the influence of mechanical artifacts affecting corticospinal mapping should be evaluated and removed to increase the robustness of the method and make it feasible to be applied in a realistic scenario.

In the future, corticospinal mapping may be combined with explicit models of the composite musculo-skeletal system. This will enable extracting whole-limb mechanical information from decoded muscle primitives, as previously proposed in, Sartori et al. (2013, 2016, 2017). This novel approach may open new avenues for the clinically viable interfacing with an individual's nervous system and the concurrent reconstruction of the intended musculoskeletal function. This methodology has the potential of, in the future, establishing man-machine interfaces that are robust and intuitive.

## Author contributions

AU: processed and analyzed the data and wrote the manuscript; MS: provided the tools for muscle primitives extraction, supervised the analyses and collaborated with the writing of the paper; JA, DF, and MS: discussed the results and implications of the experimental analysis and commented on the manuscript at all stages.

### Conflict of interest statement

The authors declare that the research was conducted in the absence of any commercial or financial relationships that could be construed as a potential conflict of interest.
